# Respiratory entrainment of the locus coeruleus modulates arousal level to avoid physical risks from external vibration

**DOI:** 10.1038/s41598-023-32995-6

**Published:** 2023-05-01

**Authors:** Masami Iwamoto, Shogo Yonekura, Noritoshi Atsumi, Satoko Hirabayashi, Hoshinori Kanazawa, Yasuo Kuniyoshi

**Affiliations:** 1 Human Science Research-Domain, Toyota Central R &D Labs., Inc., 41-1 Yokomichi, Nagakute, Aichi 480-1192 Japan; 2 Intelligent Systems and Informatics Laboratory, Mechano-Informatics Department of Graduate School of Information Science and Technology, The University of Tokyo, 7-3-1, Hongo, Bunkyo-ku, Tokyo 113-8656 Japan

**Keywords:** Computational biology and bioinformatics, Neuroscience, Risk factors

## Abstract

Slow rocking chairs can easily put people to sleep, while violent shaking, such as during earthquakes, may lead to rapid awakening. However, the influence of external body vibrations on arousal remains unclear. Herein, a computational model of a locus coeruleus (LC)-norepinephrine (NE) system and cardio-respiratory system were used to show that respiratory entrainment of the LC modulates arousal levels, which is an adaptation to avoid physical risks from external vibration. External vibrations of sinusoidal waves with different frequencies ranging from 0.1 to 20 [Hz] were applied to the LC based on the results of previous studies. We found that respiratory entrainment of the LC decreased the breathing rate (BR) and heart rate (HR) to maintain the HR within its normal range. Furthermore, 1:1 phase locking enhanced arousal level while phase-amplitude coupling decreased it for larger vibration stimuli. These findings suggest that respiratory entrainment of the LC might automatically modulate cardio-respiratory system homeostasis and arousal levels for performance readiness (fight/flight or freeze) to avoid physical risks from larger external vibrations.

## Introduction

Human emotions are influenced by external vibration. Rocking chairs with slow velocities easily put people to sleep and make them comfortable, while violent shaking, such as during earthquakes or turbulence, lead to rapidly awakening and can cause fright. Vehicles or riding game machines with vibrations may elicit feelings of fun, comfort, or sleepiness^1–3^. External body vibrations influence multiple human physiological parameters as well as the arousal level. Previous studies have indicated that whole-body vibration regulates cardio-respiratory system parameters, such as heart rate (HR) and breathing rate (BR) as well as motor or cognitive performance^4,5^ , thereby influencing arousal or sleep conditions in humans^1,2,5^.

External body vibrations with particular frequencies influence arousal. Body vibrations with frequencies ranging from 10 to 20 [Hz] decrease arousal and induce sleepiness^2,5^, while those $\le$ 0.5 [Hz] caused motion sickness with various symptoms, including drowsiness, nausea and dizziness^6^. In particular, lateral motion with frequencies of 0.2–0.4 [Hz] easily caused motion sickness^1^. In motion or simulation sickness and cybersickness with a syndrome similar to that of motion sickness, arousal levels were estimated to be enhanced^3,7^. However, there have been some contradictory reports on the matter. Body vibrations with frequencies of 10–20 Hz disturbed sleepiness^8^, while those with frequencies of 0.25 [Hz] induced sleepiness^9^. Therefore, how body vibrations change arousal in people remains unclear.

Many neurophysiological and psychophysiological studies have examined the mechanisms of arousal. In mammals, the locus coeruleus (LC) is the main source of cortical norepinephrine (NE), a modulatory neurotransmitter involved in sleep-waking and arousal that plays a significant role in regulating cognitive and attentional function^10^. When carbon dioxide (CO$_2$) levels rise, LC increases inspiratory drive as it receives inspiratory-associated activity patterns from the respiratory centre. This process can lead to greater excitatory input with a faster BR and abnormal respiratory patterns^11^. Thus, respiration may affect arousal and cognition via the LC^12^. It is well established that arousal increases pupil size, HR and skin conductance^13,14^. Thus, the LC is strongly related to cardio-respiratory activity. The LC-NE system is also influenced by external body vibration. Vibrational sinusoidal stimulations with roll tilt of mammalian bodies are known to stimulate vestibular sensory organs and then induce LC activity that is physically synchronised with the original vibrational inputs^15,16^. Thus, external body vibration may modulate LC activity and physiological parameters of the cardio-respiratory system.

Entrainment of nonlinear oscillators to an external force can be found in biological, chemical, as well as engineering systems^17,18^. Circadian rhythm and exogenous pace-maker control of the heart via entrainment have been well documented^18^. Physiological oscillations, including heartbeat and respiratory rhythm can be synchronised to appropriate external or internal stimuli^19^. Evidence suggests that the activity of a specified neuronal group is entrained by respiration or by that of another neuronal group. LC neurons are entrained by respiration^20,21^, with low-frequency oscillations in the amygdala entraining hippocampal high gamma activity^22^. The human body is assumed to have many biological oscillator ensembles, including the LC-NE, respiratory and heartbeat system. The mechanism through which nonlinear oscillators are entrained by an external force or stimulation can be elucidated through computational models of coupled oscillators^17,23,24^. Mathematical models of dynamic systems using coupled autonomous oscillators mimicking brain and respiratory or cardio-respiratory systems have been employed to investigate the mechanisms of attention and to estimate emotion recognition. Such models have revealed a positive relationship between attention state and tonic LC frequency or recognition accuracy for the circumplex model of affect according to arousal and valence^21,25^. However, there are no models that describe the detailed interactions among brain activities, such as that between the LC, cardio-respiratory activity and external body vibration.

In this study, we developed a computational model for predicting arousal level according to the interaction of the LC-NE system with the cardio-respiratory system, modelled using the Hodgkin–Huxley (HH) or FitzHugh–Nagumo (FHN) neuron models that represent the biophysical characteristic of cell membranes. We then investigated the relationship between external body vibration and arousal level. Finally, we discussed the mechanism underlying changes in arousal level, BR and HR induced via external body vibration.

## Results

### LC-respiratory entrainment elicited by body vibrations

**Figure 1:**
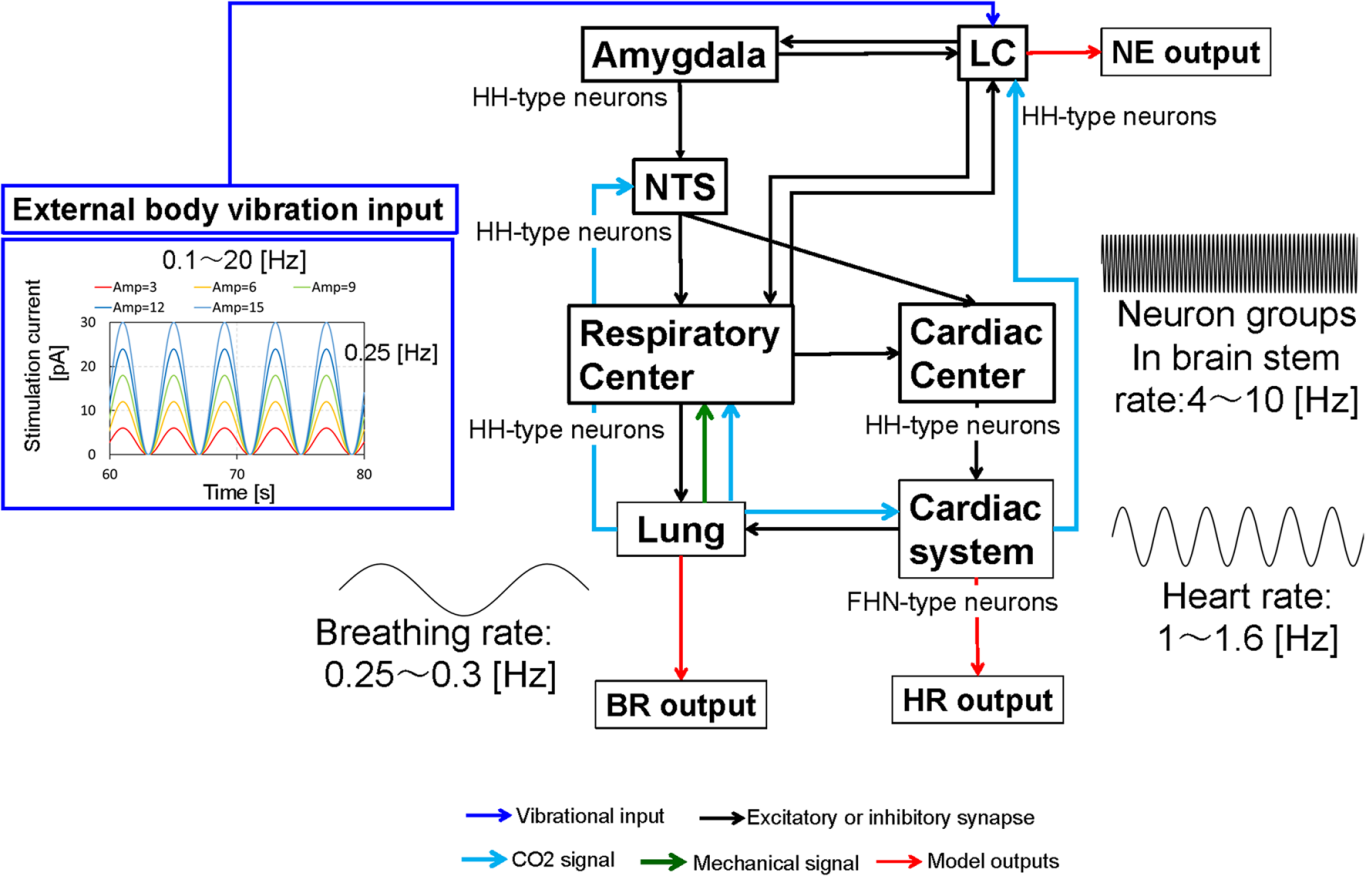
An illustration of the computational model for predicting arousal levels following external body vibration. Vibrational input, excitatory or inhibitory synaptic flows, CO$_2$ signal, mechanical signal and model outputs are depicted in blue, black, light blue, green and red, respectively. External body vibration inputs were provided by sinusoidal waves with different amplitudes and frequencies. Amygdala, LC, NTS, respiratory and cardiac centres were modelled using the Hodgkin–Huxley (HH)-type neurons, while the cardiac system was modelled using the FitzHugh–Nagumo (FHN)-type neurons. The models included different neural oscillators, wherein the default oscillatory frequencies for breathing rate (BR), heart rate (HR), and neuron groups in the brain stem were 0.25–0.3 [Hz], 1.0–1.6 [Hz] and 4–10 [Hz], respectively. BR, HR and NE-modulated conductance from the LC to the cortex, representing the arousal level were calculated as model outputs.

**Figure 2:**
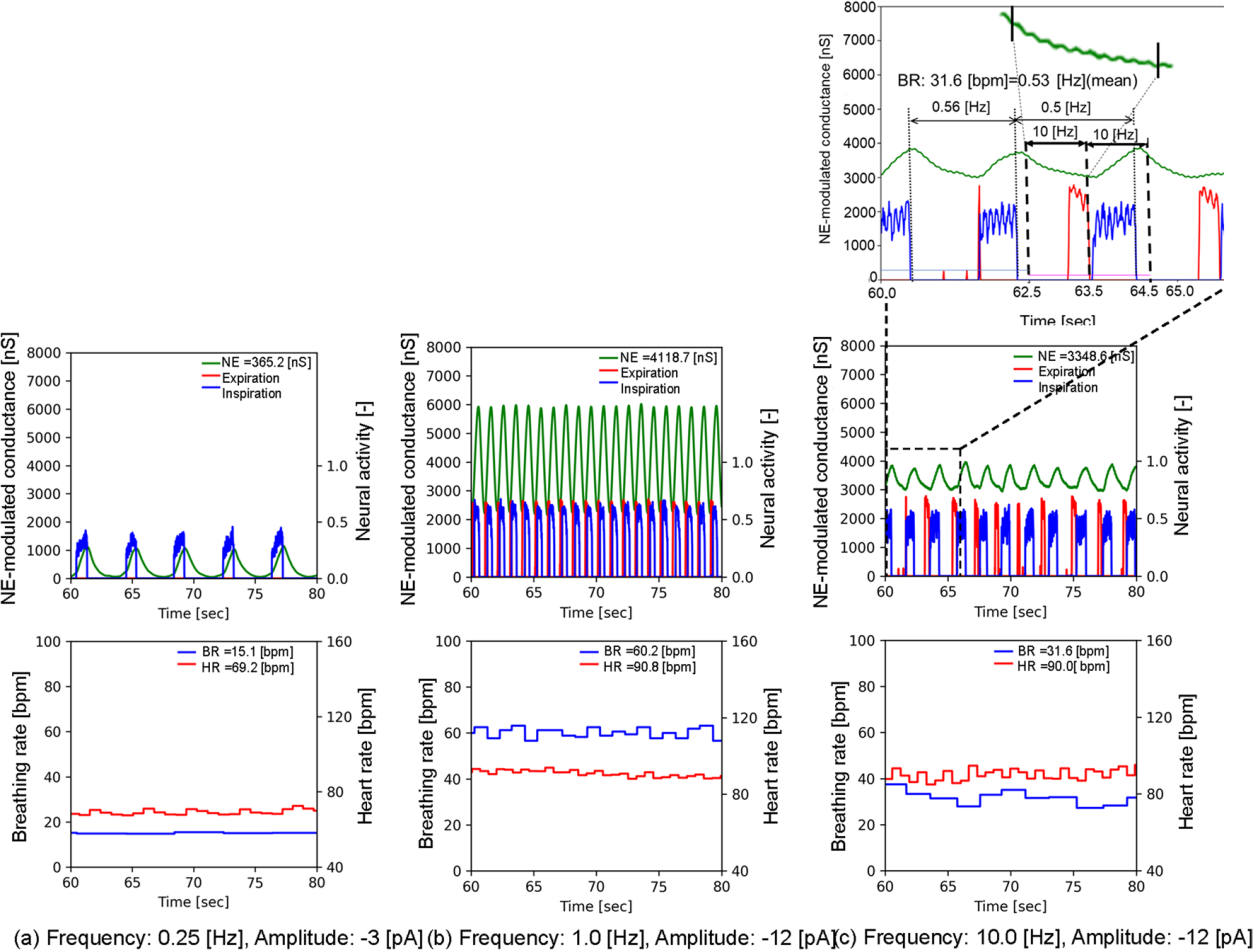
Three examples of LC-respiratory entrainment observed during the period ranging from 60 to 80 [s] in condition 1. In the upper part, the NE-modulated conductance, inspiration neuronal activity (diaphragm activity) and expiration neuronal activity (abdominal muscle activity) are shown in green, blue and red, respectively, while, in the lower part, the BR and HR are respectively shown in blue and red. (**a**) Vibration input with a frequency of 0.25 [Hz] and an amplitude of − 3 [pA], (**b**) vibration input with a frequency of 1 [Hz] and an amplitude of − 12 [pA], (**c**) vibration input with a frequency of 10 [Hz] and an amplitude of -12 [pA]. (**a**) and (**b**) demonstrate 1:1 phase locking, while (**c**) demonstrates PAC (phase-amplitude coupling).

Figure 1 shows a computational model for predicting arousal levels following external body vibration. In a case without any stimulation, NE-modulated conductance, that is, the arousal level, was 60 [ns], while the BR and HR were 16 and 68 [bpm], respectively. The values of BR and HR were almost the same as those in healthy adult men in the resting state^26,27^. With external vibrations, we found many cases in which LC activity was entrained by inspiratory activity, hereafter referred to LC-respiratory entrainment, while we also found LC-respiratory entrainment with phase-amplitude coupling (PAC), described later in the case, without any stimulation, as reported in previous studies^20,21^. Figure 2 shows three examples of LC-respiratory entrainment observed during the period ranging from 60 to 80 s in short-term body vibrations (condition 1). Figure 2 shows the NE-modulated conductance (representing LC activity and arousal level), the inspiration neuronal activity (representing diaphragm activity) and the expiration neuronal activity (representing abdominal muscle activity) as well as the BR and HR. Figure 2a–c show the results of simulation at a frequency of 0.25 [Hz] and an amplitude of − 3 pA, hereafter representing a case of 0.25 [Hz]/− 3[pA], a case of 1 [Hz]/− 12 [pA] and a case of 10 [Hz]/− 12 [pA], respectively. In both the cases of 0.25 [Hz]/− 3 [pA] and 1[Hz]/− 12 [pA], we found 1:1 phase locking (synchronisation) between LC (in green) and inspiratory activity (in blue). For the case of 10 [Hz]/− 12 [pA], we found PAC, which modulated the phase and amplitude of LC activity by respiratory activity at 0.53 [Hz], similarly to what is observed in previous studies^28,29^.

### Numerical simulation of arousal level, BR, and HR elicited by short-term body vibrations

**Figure 3:**
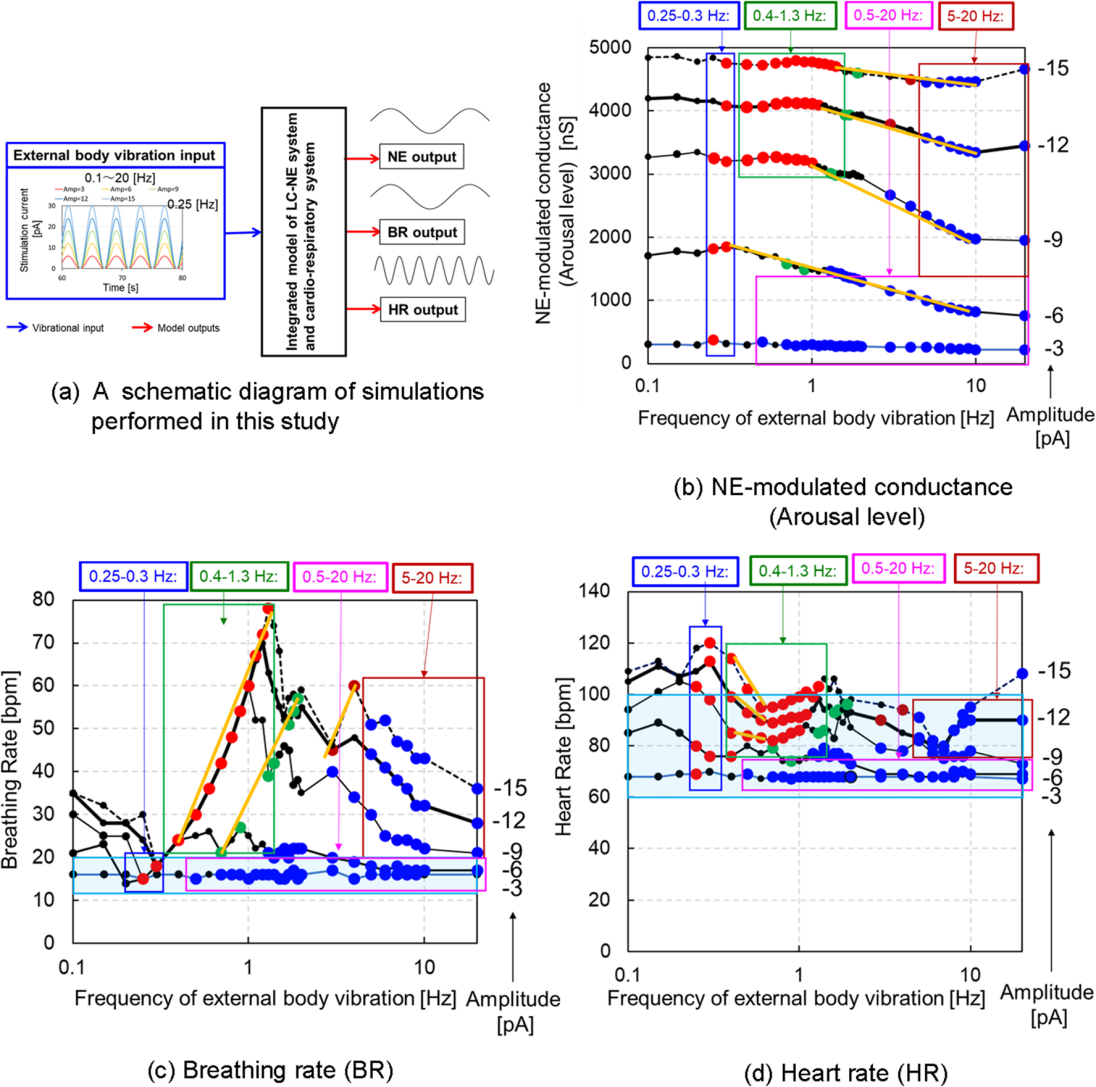
Logarithmic representation of NE-modulated conductance, BR and HR for different frequencies with different amplitudes of vibrational input in short-term body vibrations (condition 1). The red, green and brown solid circles represent the cases in which LC activity is synchronised with respiratory phrenic activity (LC-respiratory entrainment) in 1:1, 2:1 and 4:1 phase locking manner, respectively, while the blue solid circles represent the cases of LC-respiratory entrainment in the phase-amplitude coupling (PAC) manner. The black solid circles represent cases without LC-respiratory entrainment. The NE-modulated conductance of all circles was calculated as averaged values during the period ranging from 60 to 80 [s] in the autonomic breathing simulations for condition 1. (**a**) Model for predicting arousal level under assumed external body vibrations. (**b**) NE-modulated conductance is plotted along with vibration frequencies on a logarithmic scale, for different amplitudes of vibrational inputs. (**c**) BR (breathing rate) is plotted along with vibration frequencies on a logarithmic scale, for different amplitudes of vibrational inputs. The light blue flames represent the normal range of resting BR from 12 to 20 [bpm]. (**d**) HR (heart rate) is plotted along with vibration frequencies on a logarithmic scale, for different amplitudes of vibrational inputs. The light blue flames represent the normal range of resting HR from 60 to 100 [bpm]. The orange colour in (**b**)–(**d**) represents the characteristic lines to explain the mechanisms underlying the decreases in the arousal level, BR and HR observed in cases with absolute amplitude values greater than 6 [pA].

Figure 3a shows a schematic diagram of simulations performed in this study, and Fig. 3b–d show the logarithmic representation of NE-modulated conductance, BR and HR, respectively, for different frequencies with different amplitudes of vibrational input in condition 1. We noted a number of trends from the simulation results. In general, an increase in the amplitudes of the inputted sinusoidal waves tended to increase the NE-modulated conductance (arousal level), HR and BR. In a frequency range of 0.25 to 0.3 [Hz], the BR was equal to the frequency of the inputted vibration and fell within the normal range of resting BR (12–20 [bpm]) in the absolute values of the amplitude less than 12 [pA] with 1:1 phase locking, while the arousal levels were highest among all frequencies of vibration, and the HR was less than 120 [bpm] (see blue rectangles in Fig. 3b–d). At a frequency range of 0.4 to 1.3 [Hz] and an absolute value of the amplitude greater than 9 [pA], the BR was also equal to the frequency of the inputted vibration regardless of the amplitude with 1:1 phase locking. Even the BR was more than 70 [bpm], the HR almost fell within normal range of resting HR (60–100 [bpm]), while the arousal level remained high (see green rectangles in Fig. 3b–d). In a frequency range of 0.5 to 20 [Hz] and an absolute value of the amplitude lower than 6 [pA], the BR and HR almost fell within their normal ranges in the resting state, and arousal level was low with PAC (see pink rectangles in Fig. 3b–d). In a frequency range of 5–20 [Hz] and an absolute value of the amplitude greater than 9 [pA], the HR fell within the normal range in the resting state with PAC, except in a case of 20 [Hz]/− 15 [pA], while the BR totally decreased, and arousal level decreased, except in cases with a frequency of 20 [Hz] (see brown rectangles in Fig. 3b–d). The orange colours in Fig. 3b–d represent characteristic lines to explain the mechanisms of decreases in arousal level, BR and HR, with an increase in vibration frequency observed in cases with absolute values of the amplitudes greater than 6 [pA], which we later discuss. The lines in Fig. 3b indicate that the arousal level remarkably decreased with an increase in vibration frequency in cases with frequencies in the range of 1.3–10 [Hz]. The lines in Fig. 3c indicate that LC-respiratory entrainment shifted from 1:1 phase locking to 2:1 and 4:1, then moving to PAC, and BR significantly decreased with an increase in vibration frequency in cases with frequencies in the range of 1.3–20 [Hz]. The lines in Fig. 3d indicate that HR significantly decreased with an increase in vibration frequency in cases with frequencies between 0.3 and 0.5 [Hz].

NE-modulated conductance could influence the neuronal activity of the amygdala via the amygdala-LC interaction modelled for reproducing fear- and stress induced activity in the amygdala. In an amplitude of − 12 [pA], the neuronal activity of the amygdala peaked at 0.2 [Hz] and decreased with higher vibrational frequencies (Supplementary Fig. S1).

### Numerical simulation of arousal level elicited by long-term body vibrations

**Figure 4:**
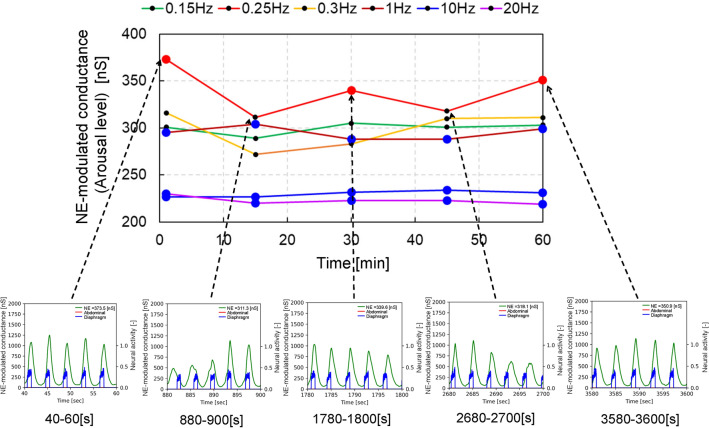
NE-modulated conductance time history for different frequencies with an amplitude of − 3 [pA] in long-term body vibrations (condition 2). The red and blue solid circles represent the cases of LC-respiratory entrainment in a 1:1 phase locking and PAC manner, respectively, while the black solid circles represent the cases without LC-respiratory entrainment. The NE-modulated conductance values of all circles were calculated as averaged values during the period of 20 [s] before each extraction point (1, 15, 30, 45 and 60 [min]) in the autonomic breathing simulations for condition 2. Five graphs in the bottom show trends of temporal change in NE-modulated conductance (green lines) and inspiration neuronal activity (blue lines) during the period of 20 [s] just before each extraction point in condition 2.

**Figure 5:**
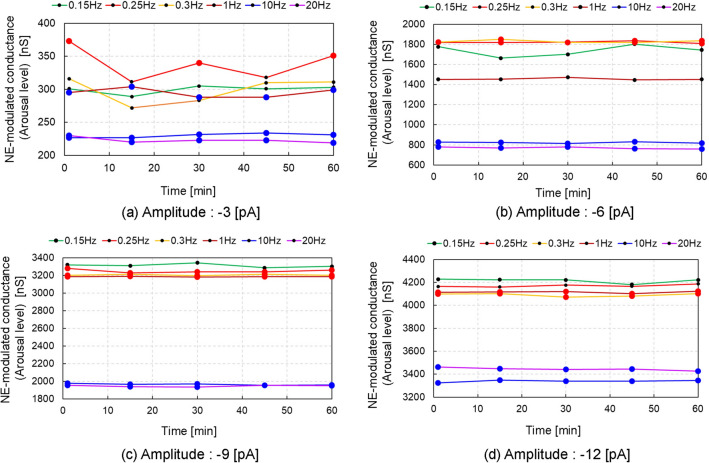
NE-modulated conductance time histories for different frequencies with varying amplitudes of vibrational input in condition 2. The red and blue solid circles represent the cases of LC-respiratory entrainment in a 1:1 phase locking and PAC manner, respectively, while the black solid circles represent the cases without LC-respiratory entrainment. The NE-modulated conductance values of all circles were calculated as averaged values during the period of 20 [s] before each extraction point (1, 15, 30, 45 and 60 [min]) in the autonomic breathing simulations for condition 2. (**a**) Amplitude of -3 [pA], (**b**) amplitude of -6 [pA], (**c**) amplitude of -9 [pA], (**d**) amplitude of − 12 [pA].

Figure 4 shows NE-modulated conductance time history for different frequencies with an amplitude of − 3 [pA] in long-term body vibrations (condition 2). The arousal level changed over time, decreasing at 15 and 45 [min] while increasing at 1, 30, and 60 [min] after stimulation onset at a frequency of 0.25 [Hz]. This kind of waves are considered to be beat frequency oscillation, which is commonly observed in acoustic and optical field, where the beating represents interference between two fields of slightly different frequencies ($\omega _1$ and $\omega _2$) and oscillates at a different frequency of $\omega _3 = |\omega _1 - \omega _2 |$^30^. In our study, the red coloured line in Figure 4 corresponds to a beat frequency oscillation waveform and $\omega _3$ was 0.00037 [Hz] in a case of 0.25 [Hz]/− 3 [pA], where BR was estimated to be equal to 0.2496 [Hz] or 0.25037 [Hz] (that is $\omega _1$), which is slightly different from the frequency of external body vibration of 0.25 [Hz] (that is $\omega _2$). 1:1 phase locking with red solid circles were observed at 1, 30 and 60 [min], where NE-modulated conductance was higher in a case of 0.25 [Hz]/− 3[pA]. In a case of an amplitude of − 6 [pA], the arousal level was highest in frequencies of 0.25 and 0.3 [Hz], but did not change over time (Fig. 5b). In cases of larger absolute values of vibration amplitudes more than 9 [pA], the arousal level was high in frequency ranging from 0.15 to 1.0 [Hz] and did not change over time under any frequency. In frequencies between 10 and 20 [Hz], the arousal level was lowest among all frequencies regardless of the vibration amplitudes. Further, 1:1 phase locking with PAC was observed over time, regardless of the vibration amplitudes. These simulation results in condition 2 are shown in Fig. 5 and Supplementary Fig. S2.

## Discussion

This study investigated the relationship between external body vibration in daily life and arousal level. Vibration can evoke multisensory signals processed by somatosensory, vestibular and visual systems. Visual and vestibular perceptual thresholds are vibration stimulations between 0.05 and 5.0 [Hz], where vestibular thresholds are between 0.1 and 1 [Hz]^31^. Recent studies on noisy galvanic vestibular stimulation (GVS) demonstrated that GVS within 0.1–10 Hz range can affect postural responses, enhance balance in healthy subjects, and influence motor performance patients with Parkinson’s disease^32^, suggesting that vestibular stimulation with frequencies lower than 10 [Hz] could modulate LC activity since the modulation of the firing rate of the vestibular system was transformed and resulted in a different firing pattern in the LC^33^. Among the four types of mechanoreceptors in somatosensory system, the Merkel’s discs, Meissner’s corpuscles and Pacinian corpuscles activate at 5–15 [Hz], 20–50 [Hz] and 60–400 [Hz], respectively. However, Merkel’s discs are cells that slowly adapt and present sensitivity to pressure, and they play a major role in the static discrimination of shapes, edges, and rough textures, which are not responsible for vibrational stimulations. Tactile perception of vibrations is performed by Meissner’s corpuscles and Pacinian corpuscles, which only activate for vibrational stimulations with frequencies $\ge$ 20 [Hz], which is beyond the scope of this study. Therefore, vibrational situations with the closed eyes were assumed to avoid the effects of visual systems, and only vestibular system was considered when determining the vibrational effect on the human body.

External body vibrations with particular frequencies change arousal levels in people. Body vibrations with frequencies ranging from 10 to 20 [Hz] were shown to decrease arousal levels and induce sleepiness^2^. Moreover, drowsiness in vehicle drivers was shown to be induced in the frequency range of 4–10 [Hz]^34^, while motion sickness was reported below 0.5 [Hz]^6^, in particular, reported at 0.2–0.4 [Hz]^1^, sometimes enhancing arousal^3,7,35^. In the present study, simulation results obtained in condition 1 demonstrated that arousal level was high in a frequency range of 0.15–0.4 [Hz], while it was low in a range of 10–20 [Hz] (Fig. 3b). In addition, neuronal activity of the amygdala peaked at a vibrational frequency of 0.2 [Hz] (Supplementary Fig. S1). The amygdala activity influences motion sickness because the lesion of the amygdala suppress the symptoms of motion sickness^36^. These results reproduced the results of above-mentioned previous studies on the relationship between external body vibrations with particular frequencies and arousal levels induced by the vibrations. However, conflicting results have also been described, as body vibrations with frequencies of 10–20 [Hz] were reported to disturb sleepiness^8^, while those with frequencies of 0.25–0.32 [Hz] induced sleepiness^9,37^. The simulation results obtained in condition 2 demonstrated that in a case of amplitude of − 3 [pA], the arousal level in a frequency of 0.25 [Hz] was highest among the other frequencies of 0.15, 0.3, 1.0, 10.0 and 20.0 [Hz], but it changed over time with beat oscillation and decreased at 15 and 45 [min] without LC-respiratory entrainment, while increasing at 1, 30 and 60 [min] with LC-respiratory entrainment after stimulation onset with a frequency of 0.25 [Hz] (Fig. 4). The results suggest that external body vibrations with a frequency of 0.25 [Hz] and a smaller amplitude could entrain respiratory activity and enhance arousal level, but the latter could fluctuate over time because the frequency of 0.25 [Hz] is very close to normal values of resting BR, and then the vibration could cause beat oscillation. The NE-modulated conductance (arousal level) fluctuated at 0.25 [Hz]; it was high a 30 and 60 [min] and low at 15 and 45 [min] (Fig. 4). These results demonstrate that the arousal levels change over time, which explains why a person might feel motion sickness at one time and sleepy at another at 0.25 [Hz]. This may also be one of the reasons for the contradiction that arousal level increases or decreases under external body vibration with a frequency of 0.25 [Hz] in motion sickness^6^, while a body vibration of 0.25 [Hz] induces sleepiness^9,37^. In contrast, the arousal level was low and changed little over time in cases with a frequency range of 10–20 [Hz] (Figure 4). Our model did not determine why body vibrations with frequencies of 10–20 [Hz] disturbed sleepiness^8^. Since arousal level could be influenced by body vibration amplitudes and the entrainment of respiratory activity, further experimental studies are needed to elucidate the relationship between external body vibrations and arousal level.

We investigated how arousal level changes along with external body vibrations using a computational model simulating the interaction of an LC-NE system with a cardio-respiratory system, which were modelled using the HH- or FHN- type neuron models. We noted many instances of LC-respiratory entrainment in cases with external body vibrations and found two types of pattern in LC-respiratory entrainment from the perspective of phase locking of nonlinear oscillators^17,23,29^. First, we found LC-respiratory entrainment, wherein external body vibration induced some n:m phase locking patterns with m inspiratory neuronal firings for n external vibrations, depending on the vibration amplitude. Entrainment is observed in a network including oscillator systems coupled to a pacemaker^17^, and the n:m phase locking is observed for external periodic forcing, depending on the forcing amplitude^38^. Vibration inputs with an increase in amplitude or frequency greater than 0.25 [Hz] increased LC-respiratory entrainment. The stimulus of increasing intensity or frequency can reinforce the phase response synchronisation in the neuronal population of weakly coupled neural oscillators^17,39^. In cases with frequencies between 0.4 and1.3 [Hz] and larger absolute values of amplitudes greater than 9 [pA], 1:1 phase locking was observed (Figs. 2b and 3). We also found LC-respiratory entrainment with 1:1 phase locking in cases where vibration frequencies are close to default oscillatory ones. 1:1 phase locking was observed in cases with frequencies between 0.25 and 0.3 [Hz], which are close to the normal BR (Figs. 2a and 3). In cases with frequencies between 1.3 and 4 [Hz] and larger amplitude absolute values greater than 9 [pA], 2:1 and 4:1 phase locking were observed (Fig. 3). Second, we found LC-respiratory entrainment with PAC, in which the amplitude of the high-frequency signal is correlated with the phase of the low-frequency signal. Cross-frequency coupling of the amplitude of high frequency activity to the phase of slower oscillations in electroencephalography (EEG) recordings has been described both in animals and in humans^28,29^. In cases with a frequency range of 0.5–20 [Hz] and absolute amplitude values lower than 6 [pA] as well as in those with frequencies between 5.0 and 20 [Hz] and absolute amplitudes values greater than 9 [pA], LC-respiratory entrainment with PAC was observed (Figs. 2c and 3). The noise-induced phenomenon, stochastic resonance (SR), occurs regardless of whether the input signal is periodic or aperiodic, weak (subthreshold) or strong (suprathreshold), and regardless of whether the system is in an excitable or oscillatory regime^40^. When the subthreshold periodic signal and noise are simultaneously imposed on the oscillators, SR could manifest itself in the form of noise-enhanced phase locking^38^. The SR profile increases according to the periodic signal intensity, changing from a bell-shaped curve to a plateau, which results in the manifestation of SR without tuning of control parameters, such as the period or the amplitude of the periodic signal and the noise strength^40^. Since the model used in this study included multiple HH-type spiking neuron groups with Gaussian noise, the noise-induced SR has a positive effect on the LC-respiratory entrainment with PAC, especially in cases with frequencies between 0.5 and 20 [Hz] and smaller absolute amplitude values of less than 6 [pA].

**Figure 6:**
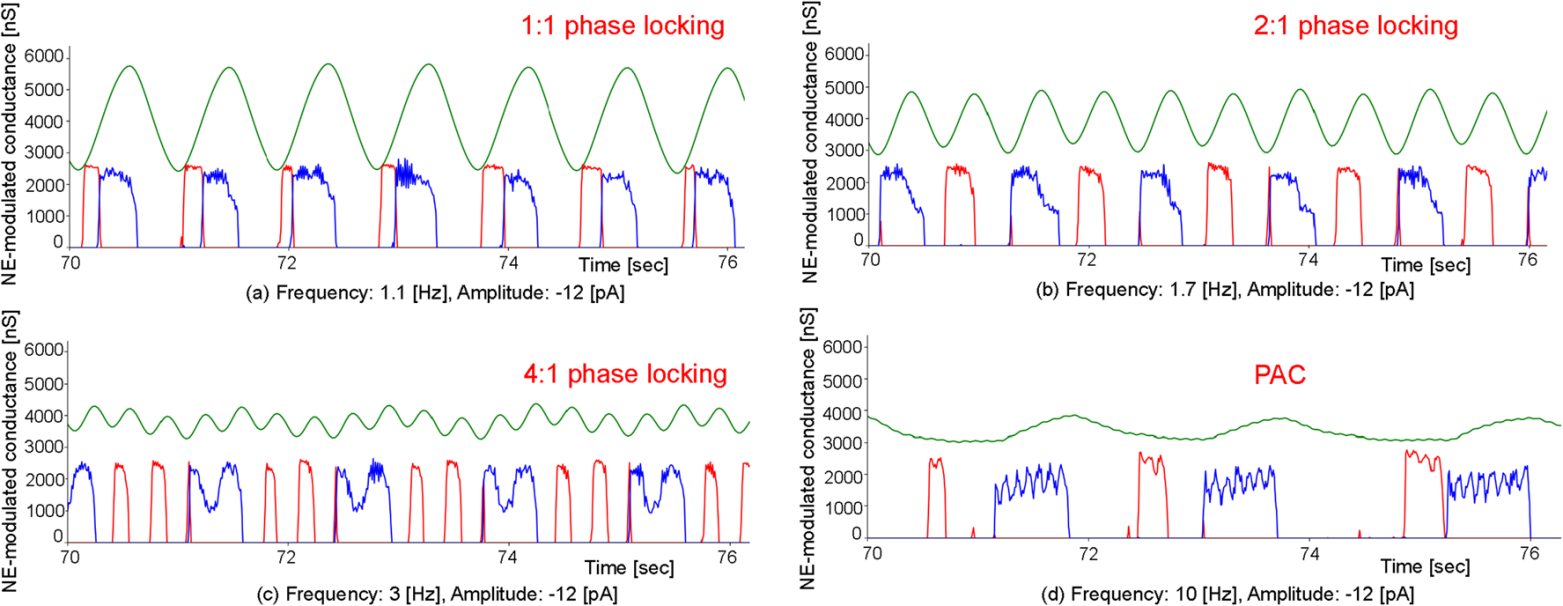
An example of mode shifts from LC-respiratory entrainment with 1:1 phase locking to PAC. Cases with an amplitude of − 12 [pA] and frequencies ranging from 1.1 to 10.0 [Hz] in condition 1 are shown. The vertical axis represents NE-modulated conductance, while the horizontal axis represents the time ranging from 70 to 76 [s]. The NE-modulated conductance, inspiration neuronal activity and expiration neuronal activity are shown in green, blue and red, respectively. (**a**) A frequency of 1.1 [Hz], (**b**) a frequency of 1.7 [Hz], (**c**) a frequency of 3 [Hz] and (**d**) a frequency of 10 [Hz].

**Figure 7:**
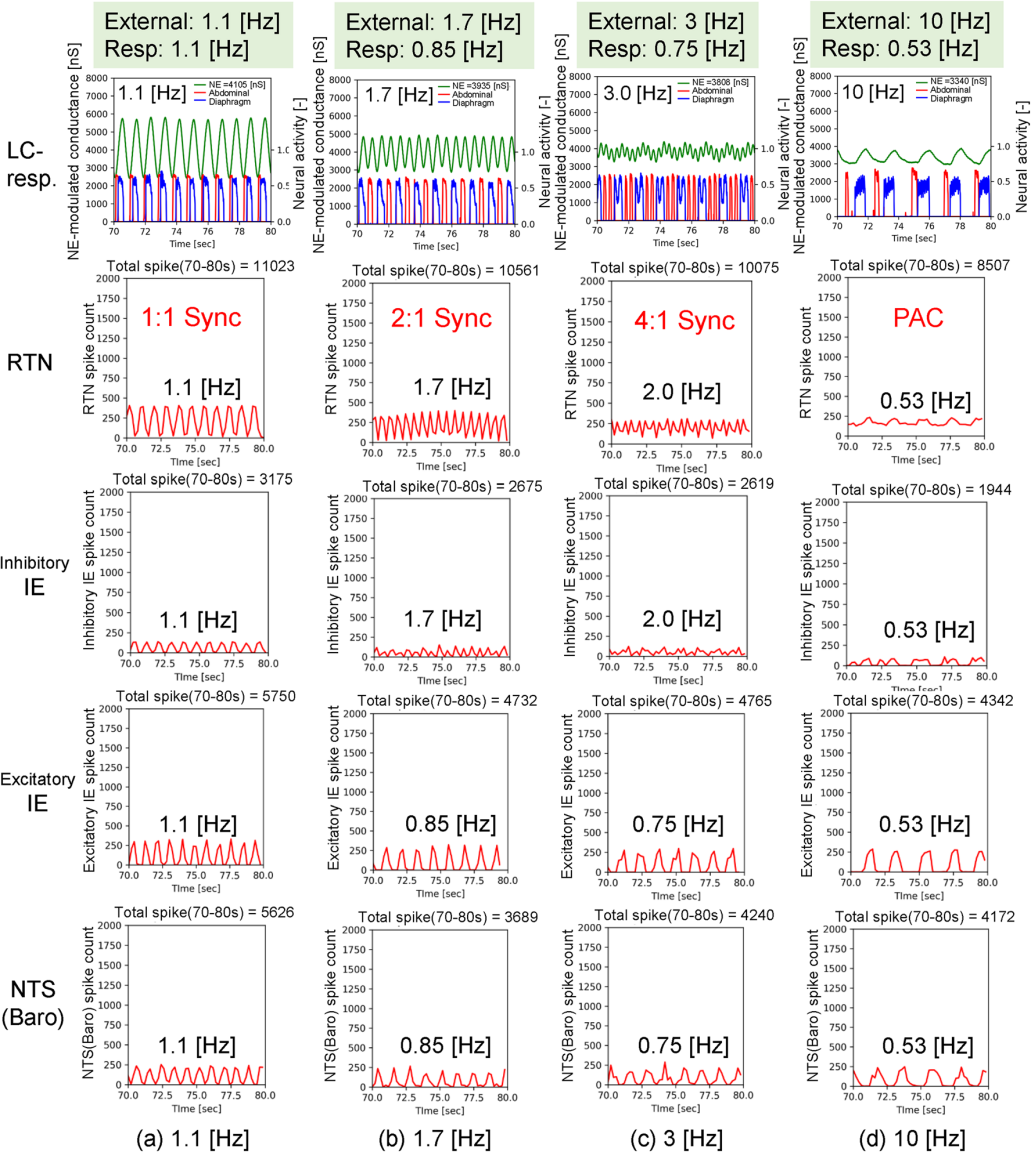
Comparisons of neuronal activity of LC-respiratory, RTN, inhibitory IE, excitatory IE and NTS (Baro) in mode shifts from LC-respiratory entrainment with 1:1 phase locking to PAC in cases with an amplitude of − 12 [pA] and frequencies ranging from 1.1 to 10 [Hz] in condition 1. The first row shows the NE-modulated conductance, inspiration neuronal activity and expiration neuronal activity from 70 to 80 [s] in green, blue and red lines, respectively. The 2nd, 3rd, 4th and 5th rows show neuronal activity of RTN, inhibitory IE, excitatory IE and NTS (Baro) with red lines from 70 to 80 [s].

**Figure 8:**
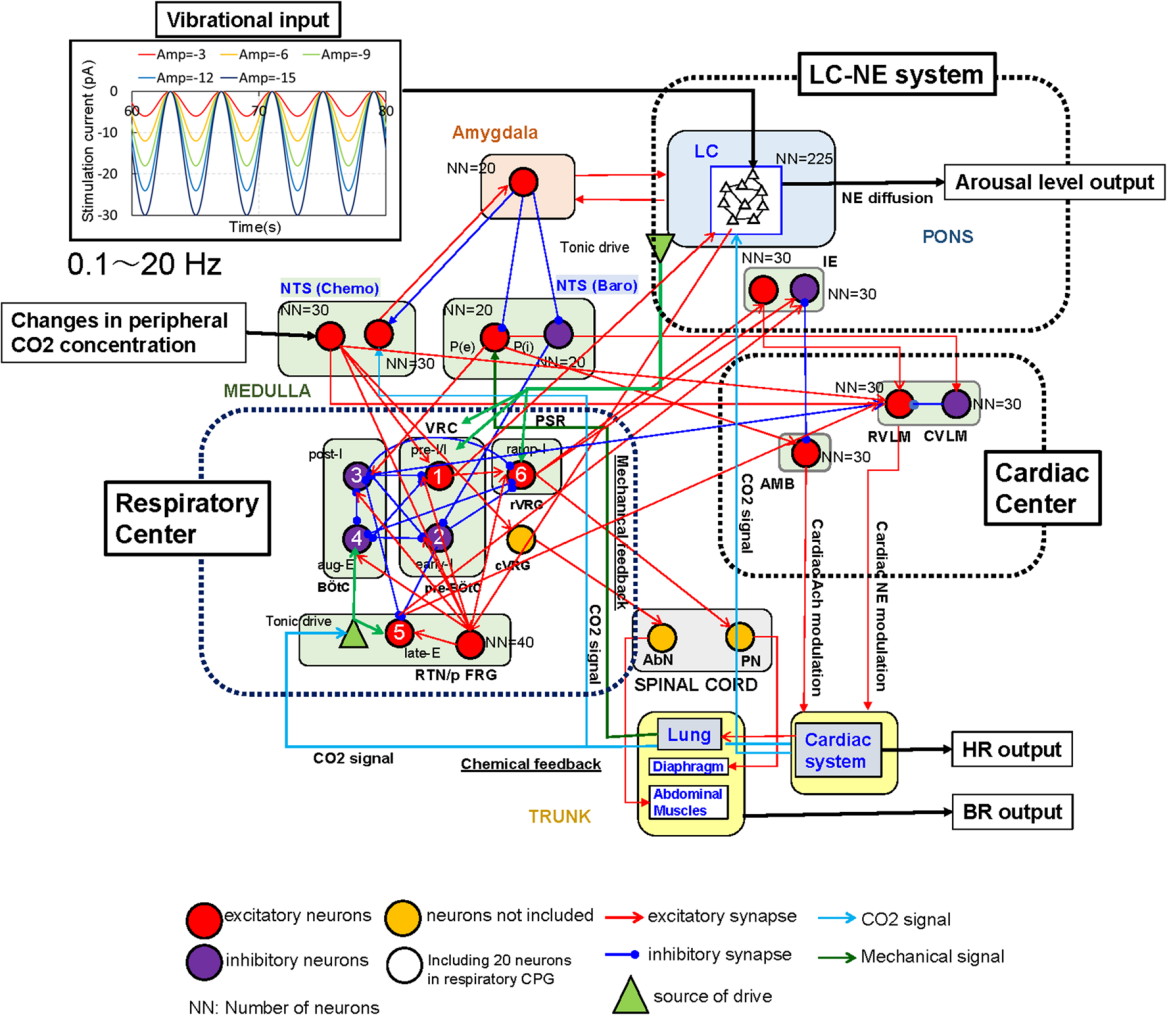
A computational model for predicting arousal levels following external stimulation. The model consists of amygdala - locus coeruleus (LC) interactions, the nucleus of the solitary tract (NTS), respiratory and cardiac centres, as well as the respiratory and cardiac systems. The respiratory centre and system comprise the closed-loop system for breathing control proposed by^41^. The closed-loop respiratory system includes two major feedback pathways from the lungs to the respiratory CPG: mechanical feedback by pulmonary stretch receptors (PSR) and chemical feedback by peripheral chemoreceptors as well as central chemoreceptors from retrotrapezoid nucleus (RTN) neurons. The cardiac centre comprises pathways that modulate heart activity: sympathetic outflow with norepinephrine (NE) modulation from the rostral ventrolateral medulla (RVLM) and parasympathetic outflow via acetylcholine (Ach) modulation from the ambiguous nucleus (AMB). Inspiratory-to-expiratory phase-spanning neurons (IE) in the pons combines the activity of the respiratory and cardiac centres. Neurons were modelled using Hodgkin–Huxley (HH)-type spiking neurons. Vibrational input is directly provided to LC. Heart rate (HR), breathing rate (BR) and NE-modulated conductance representing arousal level are outputted from cardiac system, lung and LC, respectively. cVRG: caudal ventral respiratory group.

Phase locking of oscillators is a typical phenomenon of self-organization in physical, chemical and biological systems. External periodic forcing above a threshold induces an n:m phase locking pattern with m firings for n forcing periods, depending on the forcing amplitude^38^. As shown in Fig. 3b–d, we noted different types of LC-respiratory entrainment with n:m phase locking. With an increase in the frequencies of external body vibrations from 0.4 to 20 [Hz], the mode of LC-respiratory entrainment shifted from 1:1 to 2:1 and 4:1 phase locking and then to PAC, with the BR significantly decreasing in case when the absolute amplitude values were greater than 9 [pA], which is shown as orange lines in Fig. 3(c). Fig. 6 shows an example of mode shifts from LC-respiratory entrainment with 1:1 phase locking to PAC in cases with an amplitude of − 12 [pA] and frequencies ranging from 1.1 to 10 [Hz] in condition 1, and indicates that phase locking between LC activity and inspiratory activity was found in four cases, and BR decreased with an increase in vibration frequency. Fig. 7 shows comparisons of neuronal activity of LC-respiratory, retrotrapezoid nucleus (RTN), inhibitory IE, excitatory IE, and NTS (Baro) in mode shifts from LC-respiratory entrainment with 1:1 phase locking to PAC in the same cases shown in Fig. 6. At a frequency of 1.1 [Hz], activities of RTN, NTS (Baro), excitatory IE and inhibitory IE were entrained by both LC activity and respiratory activity with 1:1 phase locking. However, at frequencies of 1.7 and 3 [Hz], activities of RTN and inhibitory IE were entrained by LC activity, while those of NTS (Baro) and excitatory IE were entrained by respiratory activity. At a frequency of 10 [Hz], activities of RTN, NTS (Baro), excitatory IE and inhibitory IE were entrained by respiratory activity, and PAC was generated. The RTN directly received excitatory synaptic signals from the LC, while the NTS (Baro) received mechanical feedback by pulmonary stretch receptors located in the lungs and transmitted information on the lung volume, that is, the BR (see Fig. 8). Therefore, the RTN was strongly influenced by LC activity, while the NTS (Baro) was strongly influenced by the respiratory activity. The excitatory IE received excitatory signals from ramp-I, while the inhibitory IE received excitatory signals from both ramp-I and late-E, which in turn directly received excitatory signals from the RTN. Thus, the inhibitory IE was influenced by RTN as compared to the excitatory IE, especially in cases with active expiration (shown as abdominal muscle activity in red lines, the 1st row of Fig. 7) to which quantal acceleration of late-E activity leads^41^.

Respiratory entrainment has a significant role on the mode shift. Respiration entrained the activity of each neuronal group, such as RTN, NTS (Baro), excitatory IE and inhibitory IE, in addition to modulating the default oscillatory frequency of each group to BR. In a case with a frequency of 1.1 [Hz], the BR was 66 [bpm], and the HR was 92 [bpm]. This demonstrates that vibrational stimulation caused respiratory entrainment with 1:1 phase locking and increased the BR up to 66 [bpm]. Somatic afferent stimulation of forelimb flexors on juvenile male rats also entrains respiratory rhythm on a 1:1 basis via the parabrachial nucleus (PB), thereby increasing the breathing frequency^42^. The PB plays an important role in controlling the cardio-respiratory systems related to blood pressure and promoting spontaneous waking in hypercapnic arousal^43–45^. Although this model showed that that respiratory rhythm entrainment could increase BR, further studies are needed to model the PB and circulatory system and investigate the effects of vibrational stimulation on breathing control and arousal. With an increase in the vibration frequency, as in a case with a frequency of 1.7 [Hz], the NTS (Baro) and excitatory IE were entrained with 2:1 phase locking, and their default oscillatory frequencies were modulated to half of the external vibration frequency. That is, the default oscillatory frequencies of NTS (Baro) and excitatory IE became 0.85 [Hz], and the BR decreased to 0.85 [Hz] (51 [bpm]). In a case with a frequency of 3.0 [Hz], the NTS (Baro) and excitatory IE were entrained with 4:1 phase locking, and their default oscillatory frequencies were modulated to a quarter of the external vibration frequency, where the default oscillatory frequencies of NTS (Baro) and IE became 0.75 [Hz], and the BR decreased to 0.75 [Hz] (45[bpm]). In a case with a frequency of 10.0 [Hz], the NTS (Baro) and excitatory IE as well as the RTN and inhibitory IE were entrained with PAC, and their default oscillatory frequencies were modulated to much lower values than the external vibration frequency, reaching 0.53 [Hz], which is about 1/20th of the external vibration frequency, with the BR decreasing to 0.53 [Hz] (32[bpm]). Similarly, respiratory entrainment decreased default oscillatory frequencies of the RVLM and the AMB, which provide respiratory-related modulation of sympathetic and parasympathetic nerves, respectively, to 0.53 [Hz], thus decreasing the HR to 90 [bpm]. The default oscillatory frequency of LC was also decreased, as was the arousal level. This is one of the reasons why arousal level remarkably decreased in the frequency range of 5–20 [Hz], shown as the orange lines in Fig. 3b.

In cases with a frequency range of 0.25–1.3 [Hz] and absolute values of amplitudes greater than 9 [pA], external body vibrations entrained the BR. Thus, the BR was equal to inputted external vibration frequency. In cases with frequencies between 0.3 and 0.5 [Hz], the HR remarkably decreased with an increase in vibration frequency, for example, the HR decreased from 113 to 93 [bpm] when the frequency increased from 0.3 to 0.5 [Hz] in cases with an amplitude of − 12 [pA]. The reason of the decrease in HR is due to the differences in neuronal activity between RVLM and AMB (see Supplementary Fig. S3). In the cases with a frequency range of 0.3–0.5 [Hz], 1:1 phase locking enhanced the neuronal activity of ramp-I and late-E in the respiratory centre with an increase in the BR and modulated the default oscillatory frequencies of RTN, NTS (Baro), excitatory IE, Inhibitory IE, RVLM and AMB to those of the external vibrations, where active expiration induced an increase in the activity of late-E and then increased the activity of AMB to a greater extent than that of RVLM. As a result, the increase in vibration frequency from 0.3 to 0.5 [Hz] remarkably decreased the HR from 113 [bpm] to 93 [bpm], which is within the normal range of resting HR, although the BR increased from 18 to 30 [bpm]. In cases with a frequency range of 0.5–20 [Hz] and absolute values of amplitudes lower than 6 [pA], LC-respiratory entrainment with PAC was observed, and BR almost fell to 15–20 [bpm] (0.25–0.33 [Hz]) in the resting state, except for cases with a frequency range of 0.5–2 [Hz] and an amplitude of − 6 [pA], while the HR was also kept within the normal values of 60–100 [bpm] in the resting state, except for a case with 20[Hz]/− 15[pA], even at larger frequencies. As mentioned previously, the noise-induced SR has a positive effect on the LC-respiratory entrainment with PAC and may stabilise the BR and HR within their normal ranges in the resting state. Previous studies have demonstrated the potential benefit of using the noise-induced SR, in which an auditory noise significantly improves the balance control of participants with lower balance control ability^46^. Therefore, LC-respiratory entrainment might have a role in maintaining homeostasis of the cardio-respiratory system.

At a frequency of 1.1 [Hz] and an amplitude of − 12 [pA], the BR was 66 [bpm], which corresponds to a very high value. A BR greater than 60 [bpm] is abnormal and life-threatening for adults. Even in new-borns, whose average resting BR lies between 30 and 60 [bpm], it is abnormal^27^. Although BR has not been sufficiently recorded as a vital sign in hospital wards, a BR of over 24 [bpm] is likely an indication of a critical illness^47^. According to the Patient at Risk scores, patients who respond to voice have BR and HR of 30–39 [bpm] and 115–129 [bpm], respectively, whereas for patients that are in pain or unresponsive, the BR and HR are greater than 40 [bpm] and 130 [bpm], respectively^48^. An increase in the resting BR and HR serves as alternative indices for measuring life-threatening risks, because hypovolemia induced by bleeding causes an increase in sympathetic nerve activity, leading to an increase in the BR and HR. In addition, BR increased up to 60 [bpm] during exercise using muscle activity such as running and high intensity internal training^49^. Since the vibration stimulation can increase muscle activity to stabilize the human posture, it may increase BR to higher values.

Thus, when the vibration frequency increases to values greater than 1 [Hz] (60 [bpm]), wherein the BR is abnormal, a strategy is required to avoid a life-threatening situation.The mode shifts from LC-respiratory entrainment with 1:1 phase locking to PAC worked well to decrease the BR and HR, and then decrease the arousal level. For instance, in cases with a vibration amplitude of − 12 [pA], an increase in vibration frequency from 1.1 to 10 [Hz] decreased the BR from 66 to 32 [bpm] and the HR from 92 to 90 [bpm], while in cases with a vibration amplitude of − 9 [pA], the BR decreased from 52 to 22 [bpm] and the HR from 88 to 78 [bpm], thus reducing the physical risks. Previous studies have demonstrated that the mode shift from 1:1 to 2:1 phase locking has some benefits for enhancing the auditory sensitivity and selectivity of male mosquitoes who detect flying females^50^ as well as for enhancing the salience of weak signals for high-power lasers in a technique known as injection-locking^18,50^. Therefore, our finding on the mode shift in cardio-respiratory system modulation might represent a biological benefit of entrainment, although further neurophysiological studies are needed to confirm it.

Although previous research has shown that the external body vibrations within a frequency range of 0.1–20 [Hz] investigated in our study may pose physical risks, we did not find any study that directly investigated the effects of the earthquakes on human body. The ground shaking due to earthquakes causes buildings to vibrate at frequencies in a range of approximately 0.1 to 30 [Hz]^51^, and it is estimated that the human body experiences the same frequencies of vibrations during the earthquakes. Whole-body vibrations with a frequency range of 1–8 [Hz] cause health risks for drivers of forestry machines^52^, while those with frequency range of 2–8 [Hz] tend to decrease their operating performance^53^. Further, whole-body vibrations with a frequency range of 4–10 [Hz] were shown to induce drowsiness in vehicle drivers^34^. Based on our results from larger vibration stimuli, the BR and HR increased with greater vibration stimuli, but the LC entrained the respiration, which in turn entrained neuron groups within the brainstem. This allowed the LC-respiratory system to increase or decrease the BR at a frequency range of 0.25–1.3 [Hz] or 1.3–10 [Hz], respectively, while keeping the HR within the normal range in the resting state so as to avoid physical risks resulting from larger external vibration. As a result, 1:1 phase locking enhanced arousal levels to generate performances, such as the fight or flight at 0.25–1.3 [Hz], whereas PAC decreased arousal levels to generate performances, such as freeze or unresisting at 1.3–10 [Hz] for larger external vibration. Thus, we hypothesise that LC-respiratory entrainment has a role in the automatic modulation of cardio-respiratory system homeostasis and the arousal level for performance readiness (fight/flight or freeze) to avoid physical risks resulting from larger external vibration.

This study has some limitations. First, we used the assumed current values of − 3 to − 15 [pA] to represent the amplitudes of vibration inputs to LC. Further neurophysiological studies are necessary to determine how large the electrical stimulus to LC is when human body sustains external vibrations. Second, the arousal level in motion sickness is different depending on the direction of body vibration^1^. The vibrational sinusoidal stimulations to vestibular sensory organs for inducing LC activity were performed for left and right rotations (roll) around the longitudinal direction^15,16^, while the vibrational sinusoidal stimulations to vestibular sensory organs for inducing responses of caudal medullary raphe neurons were performed for forward and backward rotations (pitch) around the lateral direction^54^. However, our model cannot set the direction of body vibration to the longitudinal, lateral and vertical directions because it did not include caudal medullary raphe neurons or other related neurons. Further experimental and computational studies are necessary to investigate effects of vibration direction on the arousal level. Finally, the integrated model of the LC-NE system and cardio-respiratory system included two neuronal oscillator groups, except the respiratory system, that is, the cardiac system and neuron groups in the brainstem, which have default oscillatory frequency ranges of 1–1.6 [Hz], and 4–10 [Hz], respectively. The system could have nonlinear oscillator ensemble to create complex phase locking patterns^24^. However, we could not determine how default oscillatory frequency ranges of the two neuronal oscillators are related to the LC-respiratory entrainment we found in this study. This might be because the respiratory system can have a much stronger entrainment effect than the other neuronal oscillators. Future studies should investigate the mechanism of LC-respiratory entrainment using a computational model including more neuronal oscillators simulating various ones found throughout the brain and body.

## Methods

### Coupling of the LC-NE and cardio-respiratory systems

Figure 8 illustrates the model used in this study. We modelled the amygdala, RTN, RVLM, caudal ventrolateral medulla (CVLM) and AMB using 20, 40, 30, 30 and 30 HH-type spiking neurons, respectively. Baroreceptor-related neurons in the NTS (NTS Baro) and IE include 20 and 30 sets of excitatory and inhibitory HH-type neurons, respectively. Chemoreceptors in the NTS (NTS Chemo) include two groups of 30 HH-type neurons: one receives CO$_2$ signals from the lungs and excites or inhibits the amygdala^55,56^, and the other comprises second-order peripheral chemoreceptors, which have a direct projection to the RTN, preI/I (pre-BötC) and RVLM^57^. Since the number of HH-type spiking neurons may influence the simulation results^58^, we performed parametric simulations using different number of HH-type spiking neurons. The default numbers of HH-type spiking neurons for the amygdala, RTN, RVLM, CVLM, AMB, NTS Baro, NTS chemo and IE were set to 20, 40, 30, 30, 30, 20, 30 and 30, respectively. We simulated 0.5, 1.5 and 2 times of the default numbers of HH-type spiking neurons, for instance, in case of 2 times, they were set to 40, 80, 60, 60, 60, 40, 60 and 60, respectively. The parametric simulation results demonstrate that smaller numbers of HH-neurons had different values in BR and HR in comparison with the default numbers (Supplementary Fig. S4).

Each neuron can be fundamentally represented using the following equations.

1 $$\begin{aligned} C \frac{dV}{dt }= & {} -\overline{g}_K m_K^4 (V-E_K) - \overline{g}_{Na} m^3_{Na} h_{Na} (V-E_{Na}) \nonumber \\{} & {} {} -g_L (V - E_L) - I - \sqrt{2 D} \xi (t), \end{aligned}$$

where *C* is the membrane capacitance as $C = 36$ [pF], while $\overline{g}_K, \overline{g}_{Na}$ and $g_L$ are the peak conductances of potassium and sodium as well as the leak conductance (Leak), respectively. $\overline{g}_K = 250$ [nS], $\overline{g}_{Na} = 400$ [nS] and $g_L = 6$ [nS], while $E_K, E_{Na}$ and $E_L$ represent the reversal potentials of potassium, sodium and Leak, respectively. $E_K = -94$ [mV], $E_{Na} = 55$ [mV] and $E_L = -60 \pm 1.2$ [mV], provided a Gaussian distribution with a mean of − 60 and variance of 1.2. $m_K$, $m_{Na}$ and $h_{Na}$ are gating parameters that depend on the membrane variable V.

For $$z = {K, Na}$$,

2 $$\begin{aligned}{} & {} \tau _{m z} d m_z/dt = m_{\infty z}(V) - m_z, \end{aligned}$$

3 $$\begin{aligned}{} & {} \tau _{h z} d h_z/dt = h_{\infty z}(V) - h_z \end{aligned}$$

The constants were determined based on^59^, as follows:

4 $$\begin{aligned}{} & {} m_{\infty K} = \alpha _{\infty K} / (\alpha _{\infty K} + \beta _{\infty K}), \end{aligned}$$

5 $$\begin{aligned}{} & {} \alpha _{\infty K} = 0.01 (V + 44.0) / (1.0 - \exp (-(V + 44.0)/5.0)), \end{aligned}$$

6 $$\begin{aligned}{} & {} \beta _{\infty K} = 0.17 \exp (-(V + 49.0)/40.0), \end{aligned}$$

7 $$\begin{aligned}{} & {} m_{\infty N_a} = 1.0/(1.0 + \exp (-(V + 34.0)/7.8)), \end{aligned}$$

8 $$\begin{aligned}{} & {} h_{\infty N_a} = 1.0/(1.0 + \exp ((V + 55.0)/7.0)). \end{aligned}$$

The time constants were set as follows:

9 $$\begin{aligned}{} & {} \tau _{mK} = 3.5 / \cosh ((V + 40.0)/40.0), \end{aligned}$$

10 $$\begin{aligned}{} & {} \tau _{h Na} = 8.456 / \cosh ((V + 67.5)/12.8), \end{aligned}$$

where $\tau _{mK}$ and $\tau _{h Na}$ are in [ms]. $m_{Na}$ was assumed to be $m_{\infty N_a}$, and the time constant was assumed to be $\tau _{m Na}=0$ based on a previous result^60^. $\xi (t)$ is Gaussian noise, and *D* is noise strength, with $D=20$ [pA]. Equation (1) was updated using the Euler method. In this study, the time step was set as 1.0e−4 [s].

The LC was modelled using a $15 \times 15$ excitatory neural network with an HH-type equation, as follows:

11 $$\begin{aligned} C \frac{dV}{dt}= & {} -\varphi g_L(V-E_L) - \overline{g}_K m_k^4 (V-E_K) \nonumber \\{} & {} {} - \overline{g}_{Na} m_{Na}^3 h_{Na} (V-E_{Na}) - I - \sqrt{2 D} \xi (t) \nonumber \\{} & {} {} -\varphi I_{syn}^E -I_{NE}^I - I_{gap}, \end{aligned}$$

where $I_{syn}^E$ is the excitatory synaptic input, and $I_{NE}^I$ represents the NE interaction, as described later. $I_{gap}$ is the electrical current based on the gap-junction from the eight neighbouring neurons and is computed as $I_{gap}=\sum _j g_{gap} (V_i - V_j)$. $\varphi$ is the modulating term reflecting CO$_2$ concentration (pCO$_2$) in the blood, and was described according to a previous study^61^, as follows:

12 $$\begin{aligned} \varphi = 1- \frac{1}{1+(s_{1/2}/s)^{h_s}}, \end{aligned}$$

where we assumed $s_{1/2} = 10, h_s = 5$ and $s=pCO_2/760.0\times 10^{-2}$. We set $C = 25$[pF], $\tau _{mK} = 1.75/cosh((V+40))/40)$ [ms] and $g_{gap} = 0.005$ [nS] to reduce the firing rate of spontaneous ignition to around 1 [Hz]. Although Quintero et al.^61^ used an HH-type model that included $Ca^{2+}$ channels, we used one without them for simplicity. NE emitted by the excitatory firing of LC neurons is diffused to cognitive neurons and modulates the activation gain $g_{NE}$. The modulatory dynamics for $g_{NE}$ are described using the second-order delay system, as follows:

13 $$\begin{aligned}&\tau _0 \dot{y} = -y + \sum _j \delta (t-t_j), \end{aligned}$$

14 $$\begin{aligned}&\tau _1 {\dot{g}}_{NE} = - g_{NE} + y, \end{aligned}$$

where $t_{j}$ is the *j*th firing spike, and $g_{NE}$ corresponds to the NE-modulated conductance released into the surroundings. $I_{NE}^I = g_{NE}^0 g_{NE} (V - V_{syn}^I)$. $g_{NE}^0 = 0.03\times 10^{-3}$[nS], $V_{syn}^I = -75$[mV]. The rise and decay time constants of $\tau _0$ and $\tau _1$ are 100 [ms] and 300 [ms], respectively, as previously reported^62^.

The LC in the pons supplies NE input to many regions of the cortex and the respiratory centre. The diffusion of NE in the cortex is associated with arousal; an increase in NE is accompanied by an increase of arousal level. Chemosensitivity in LC neurons is activated by an increase in CO$_2$ concentration in the blood, as the LC receives excitatory information from the respiratory CPG (central pattern generator). We found that the controller gain of the chemical feedback from the lung to the LC can influence the time-to-respond for an emotional fear- or surprise-related stimulus^63^. LC neurons also receive excitatory input from preI/I (pre-BötC) neurons, as previously shown in adult mice^11^. Since LC neuron activity is synchronized with inspiratory bursts, we modelled the connectivity from preI/I to LC with all-to-all connections. However, there is no evidence of their synaptic strengths. We set their synaptic strengths to 0.1 based on synaptic strengths for the other connectivity in respiratory CPG. In addition, the direct pathway from the LC to the RTN/pFRG (parafacial respiratory group) induces active inspiration and expiration^64^. Therefore, NE diffusion, that is, the activity of LC, indirectly influences the control of breathing. We included the amygdala-LC interaction in the model because fear- and stress-induced activity in the amygdala influences sensory brain regions through connections with the LC^65^. Furthermore, increased tonic activity in LC neurons induces anxiety-like and aversive behaviour^66^. However, we did not include the cortical and subcortical controls of breathing associated with emotion and cognition. Although the amygdala-LC interaction includes GABAergic inhibitory connections^67^ and corticotropin-releasing factor, which has an excitatory effect on LC neurons^68^ and adjusts the activity as well as reactivity of the LC-NE system^69^, we only included the excitatory interactions between the amygdala and LC in our model.

The respiratory centre and respiratory system were developed based on a mathematical model of the closed-loop system for the control of breathing proposed by^41^. Breathing or lung ventilation represents the exchange of air between the lungs and the surrounding environment, which takes place according to the rhythmic contraction of the inspiration and expiration neurons. The firing activities of the phrenic and abdominal motor neurons control the inspiration and expiration neurons, respectively, representing major outputs of the brainstem respiratory CPG, which generates respiratory oscillations. The closed-loop respiratory system includes two major feedback pathways from the lungs to the respiratory CPG, namely mechanical feedback and chemical feedback. Mechanical feedback is provided by pulmonary stretch receptors (PSR) located in the lungs, which transmit information regarding lung volume to the brainstem via the vagus nerve. Chemical feedback is assumed to be provided by changes in the peripheral CO2 concentration as a signal from the peripheral chemoreceptors to NTS Chemo^57^, which is sensitive to the levels of oxygen (O$_2$) and CO$_2$ in the blood and promotes RTN activity, thereby causing an increase in HR and BR (see Fig. 8). We adjusted the NTS Chemo parameters so that active expiration was not induced by the activity of abdominal muscles during normal breathing; however, active expiration was induced during hypercapnia according to previous results^57^. The arterial CO2 partial pressure of around 40 mmHg was provided to central chemoreceptors of the RTN during normal breathing. The central chemoreceptors of the RTN, which are highly sensitive to the CO$_2$ concentration in the brainstem, and thus modulate respiratory CPG in a CO$_2$-dependent fashion. The parameters of the respiratory centre and system were originally adjusted to the adult rat respiratory system^41^. We replaced mechanical system of the lung with a mathematical model of respiratory muscles, chest wall and lungs based on adult human subject data previously proposed by^70^. Then, we modified the parameters to adjust the adult human respiratory system as listed in Table 1.

**Table 1:** Parameters adjusted for adult human respiratory system.

	Original adult rat system^41^	Modified adult human system^70,71^
Lung elastance E [mmHg/L]	3000	3.659
Heart period $T_L$ [s]	0.167	0.833
Volume of the lung capillaries $V_c$ [L]	0.000192	0.070
Acceleration rate of the chemical reaction $\delta$ [-]	500	$10^{1.9}$
Diffusion capacity of CO$_2$ $D_C$ [L/mmHg/s]	$10^{-4}$	$7.08\times 10^{-3}$
Diffusion capacity of O$_2$ $D_O$ [L/mmHg/s]	$5\times 10^{-6}$	$3.5\times 10^{-4}$
Time constant of gating variable m2 $\tau _{m2}$ [s]	0.67	2.0
Time constant of gating variable m3 $\tau _{m3}$ [s]	0.67	2.0

The cardiac centre was developed based on the pathways via which the central nucleus of the amygdala (CeA) influences blood pressure during mental stress or anxiety, as proposed by^72^, and autonomic chronotropic control of the heart, as previously depicted^73^. During stressful conditions, the CeA may inhibit baroreceptive neurons in the NTS. This inhibition might switch off inhibitory inputs from the CVLM to the RVLM, which could activate neurons in the RVLM, leading to an increase in sympathetic outflow with NE modulation and an increase in blood pressure and HR. The AMB also receives excitatory information from the NTS, which increases parasympathetic outflow via acetylcholine (Ach) modulation of heart activity, thus decreasing HR. The cardiac system was represented using a cardiac muscle activation model with electrical activity based on the FitzHugh–Nagumo (FHN) neuron model^74^, as well as the neuromodulation of NE and Ach, which are neurotransmitters in sympathetic and parasympathetic nerves, respectively. The electrophysiological model of cardiac muscle activity is described by the following diffusion equation:

15 $$\begin{aligned}{} & {} \dot{v} = D \nabla ^2v + c(v(v-\alpha )(1-v)-w), \end{aligned}$$

16 $$\begin{aligned}{} & {} \dot{w} = b(v-dw), \end{aligned}$$

17 $$\begin{aligned}{} & {} c = \left\{ \begin{array}{ll} c_{1S} &{} (\dot{v} \ge 0), \\ c_{2S} &{} (\dot{v} < 0), \\ \end{array} \right. \end{aligned}$$

where *v* is the membrane potential of cardiac muscle cells, and *w* is the recovery variable. $c_{1S} = G_c \overline{c}_{1S}, c_{2S} = G_c \overline{c}_{2S}, \overline{c}_{1S} = 1$, and $\overline{c}_{2S} = 0.22$. $\alpha$, *b* and *d* are parameters that define the shape of *v*. We hypothesised that $G_c$ and *b* would be determined by the neuromodulation of NE and Ach, as shown in the following Eqs. (20, 21), based on Hill’s Eq.^75^.

18 $$\begin{aligned}{} & {} G_c = (c_{Max} - c_{Min})*H(p_{NE}) + c_{Min}, \end{aligned}$$

19 $$\begin{aligned}{} & {} b = (b_{Max} -b_{Min})*H(q_{Ach}) + b_{Min}. \end{aligned}$$

20 $$\begin{aligned}{} & {} H(p_{NE}) = 1/(1+(p_{NE_{mid}}/p_{NE})^{\alpha _{NE}}), \end{aligned}$$

21 $$\begin{aligned}{} & {} H(q_{Ach}) = 1-1/\left( 1+\left( q_{Ach_{mid}}/q_{Ach}\right) ^{\alpha _{Ach}}\right) . \end{aligned}$$

The HR increases with the value of parameter *b* and decreases with the value of *b*. $H(q_{Ach})$ significantly contributes to the adjustment of HR. The average HR ranges from 64 to 69 [bpm] in adult men aged 20–60 years^26^, while the average breathing rate (BR) in adults at rest is 12–20 [bpm]^27^. We set $p_{NE_{mid}}=350.0$, $q_{Ach_{mid}}=20.0$, $\alpha _{NE}=10.0$, and $\alpha _{Ach}=5.0$ to reproduce the average values of BR and HR.

In this study, we introduced inspiratory-to-expiratory phase-spanning neurons (IE), including excitatory and inhibitory neurons, into the pons to combine the activity of the respiratory centre with that of the cardiac centre. The excitatory IE was proposed by^57,76^ to receive an excitatory signal from ramp-I of the respiratory centre and project to the RVLM, providing a pontine-dependent inspiratory modulation of thoracic sympathetic nerves according to a suggestion of respiratory-sympathetic coupling based on previous experimental observation^77^. Further, the role of the excitatory IE was confirmed with additional computational and experimental studies^78^. The inhibitory IE was described to receive excitatory signals from the respiratory centre, projecting into the AMB and providing respiratory-related modulation of parasympathetic nerves based on recent neurophysiological studies^79,80^. Here, we added an excitatory projection from ramp-I neurons in the respiratory centre to the inhibitory IE in order to control how the flow of information from the NTS to the AMB is cut off during inspiration, as per^73^, as well as an excitatory projection from late-E neurons to inhibitory IE to represent hypoxia-induced transient tachycardia, as reported by^81^.

This model has several parameters; we mostly determined the parameters based on previous studies^41,60–62,70,71^, However, we used four parameters $p_{NE_{mid}}$, $q_{Ach_{mid}}$, $\alpha _{NE}$ and $\alpha _{Ach}$ to adjust the BR and HR to be 16 and 68 [bpm], respectively, which are almost the same as those in healthy adult males in the resting state, by approximately five times of trial and error.

### Experimental design

We used the developed computational model for autonomic breathing simulation under the following two types of external body vibration conditions (Fig. 1). Condition 1 included a total of 155 inputs to simulate human body vibrations via sinusoidal waves with five amplitudes (− 3, − 6, − 9, − 12 and − 15 [pA]) and 31 frequencies (0.1, 0.15, 0.2, 0.25, 0.3, 0.4, 0.5, 0.6, 0.7, 0.8, 0.9, 1.0, 1.1, 1.2, 1.3, 1.4, 1.5, 1.6, 1.7, 1.8, 1.9, 2.0, 3.0, 4.0, 5.0, 6.0, 7.0, 8.0, 9.0, 10.0 and 20.0 [Hz]) in a time period of 80 [s]. Condition 2 included a total of 24 inputs to simulate those with four amplitudes (− 3, − 6, − 9 and − 12 [pA]) and six frequencies (0.15, 0.25, 0.3, 1, 10 and 20 [Hz]) in a time period of 60 [min], during which simulation results were obtained at 1 [min] and every 15 [min], hereafter referred to as extraction points. As mentioned previously, vibrational inputs to mammalian bodies are known to stimulate vestibular sensory organs and then induce LC activity that is physically synchronised with the original vibrational inputs^15^. However, how the amplitudes of vibrational inputs are transmitted to the LC is unknown. Therefore, we used current values inputted to HH-type neurons to represent the amplitudes of the stimulations inputted to the LC. All amplitudes represent excitatory stimulation, which increased with the absolute value.

We calculated the BR, HR and NE-modulated conductance from the LC to the cortex, which represent the arousal level, activity of the inspiration and expiration neurons, as well as neuronal activity of amygdala, NTS (Baro), RTN, IE, RVLM and AMB as model outputs. Activity of the inspiration and expiration neurons ranged from 0 to 1, while neuronal activity of amygdala, NTS (Baro), RTN, IE, RVLM and AMB was calculated as neuronal spike counts. The BR, HR and NE-modulated conductance were calculated as averaged values during the period ranging from 60 to 80 [s] in the autonomic breathing simulations for condition 1 and during the period of 20 [s] before each extraction point for condition 2. We investigated the relationship between external body vibration input and model outputs of arousal level, BR, and HR using the obtained simulation results. The model was implemented using C++ language, and all simulations were performed using a computer with an Intel Xeon Gold 6242 (16C/2.8Ghz) and 384 GB Memory.

## Supplementary Information

Supplementary Figure S1.

Supplementary Figure S2.

Supplementary Figure S3.

Supplementary Figure S4.

## Data Availability

The source codes used to generate the results in this paper are available at https://osf.io/bzdtf/?view.
