# Supplementary figures and images for: Respiratory entrainment of the locus coeruleus modulates arousal level to avoid physical risks from external vibration
Source: Sci Rep. 2023 May 1;13:7069. doi: 10.1038/s41598-023-32995-6 (PMC10151378; doi:10.1038/s41598-023-32995-6)

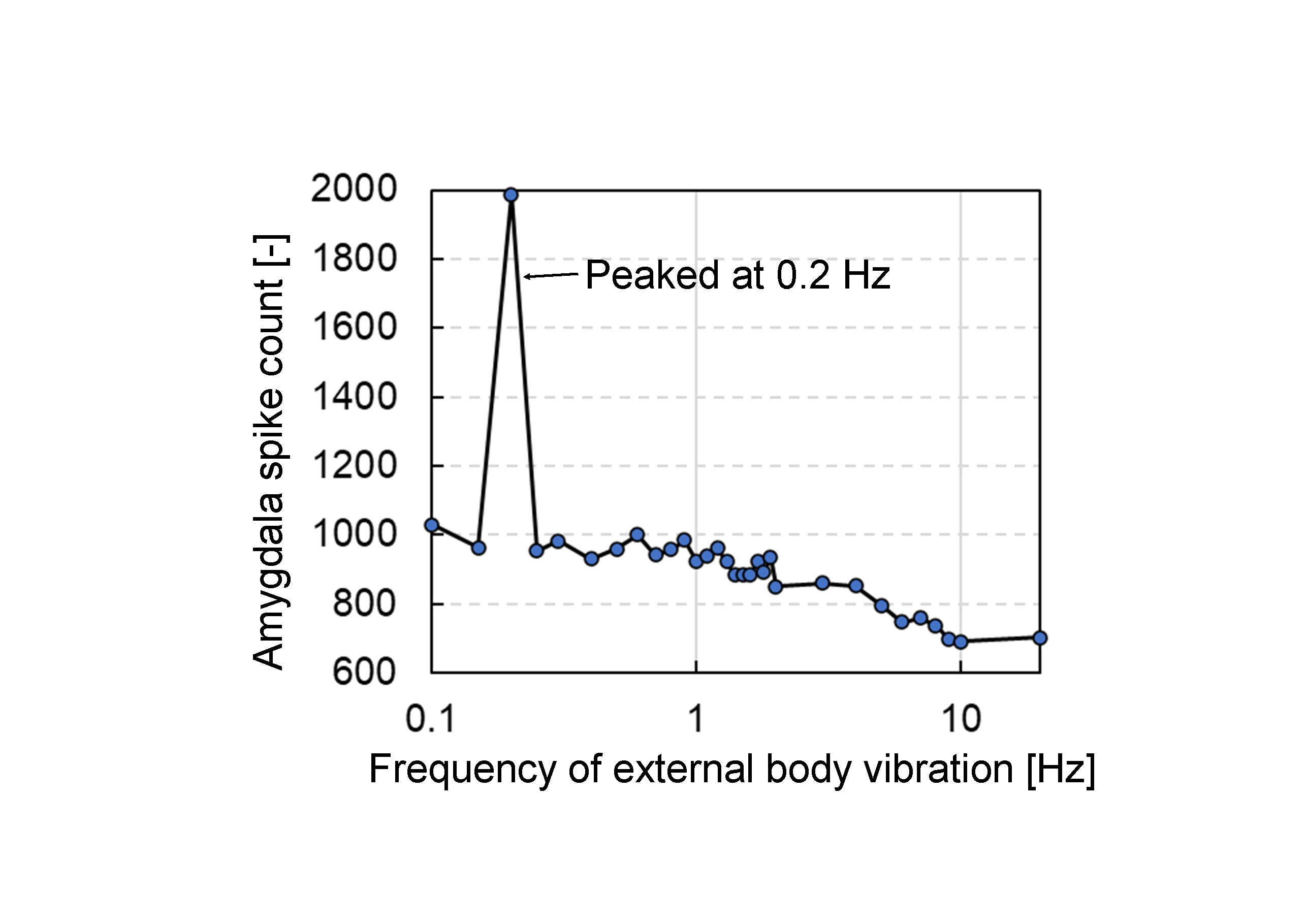

Supplement: Supplementary file 1 — Supplementary Figure S1. [file 41598_2023_32995_MOESM1_ESM.tif]

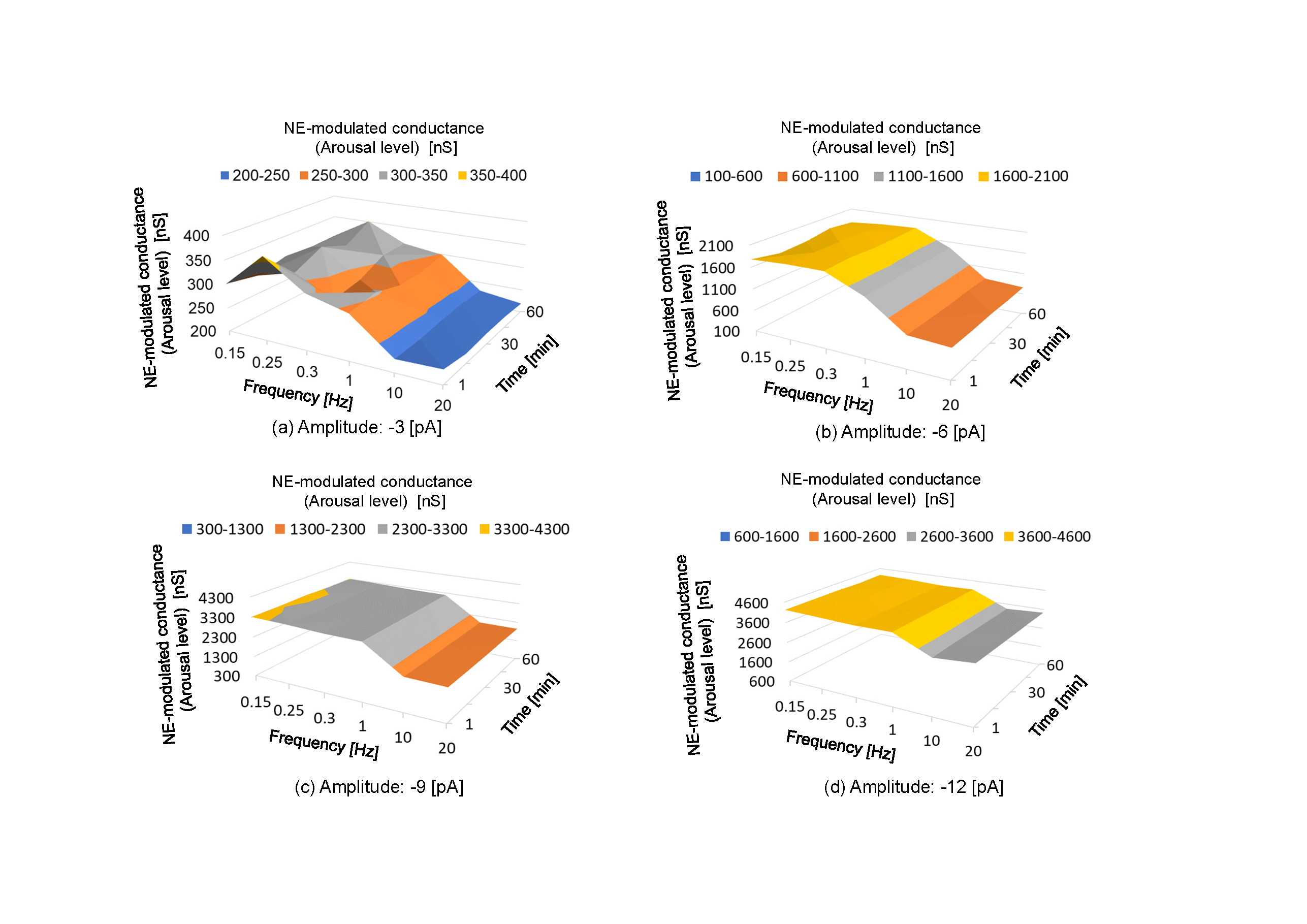

Supplement: Supplementary file 2 — Supplementary Figure S2. [file 41598_2023_32995_MOESM2_ESM.tif]

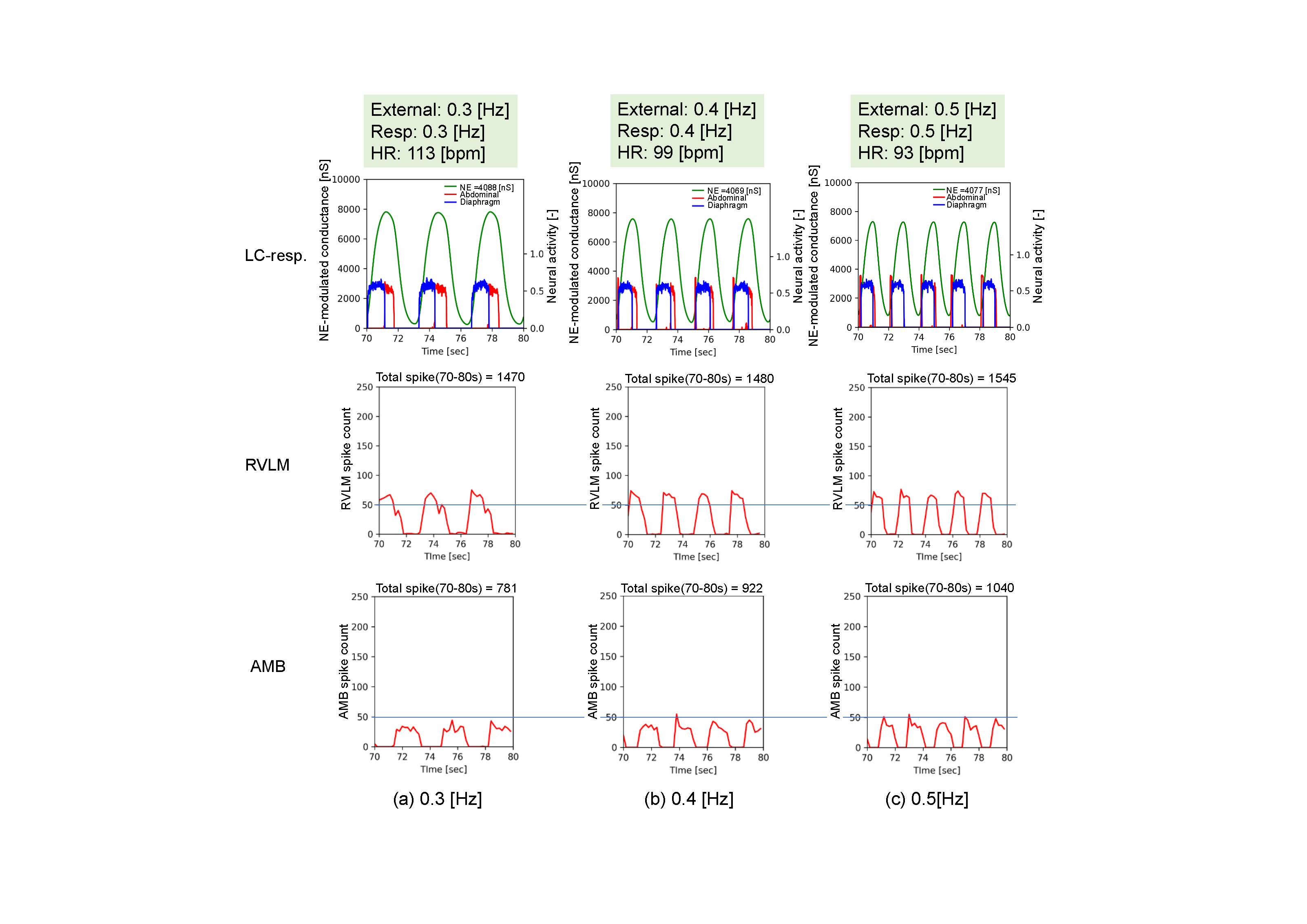

Supplement: Supplementary file 3 — Supplementary Figure S3. [file 41598_2023_32995_MOESM3_ESM.tif]

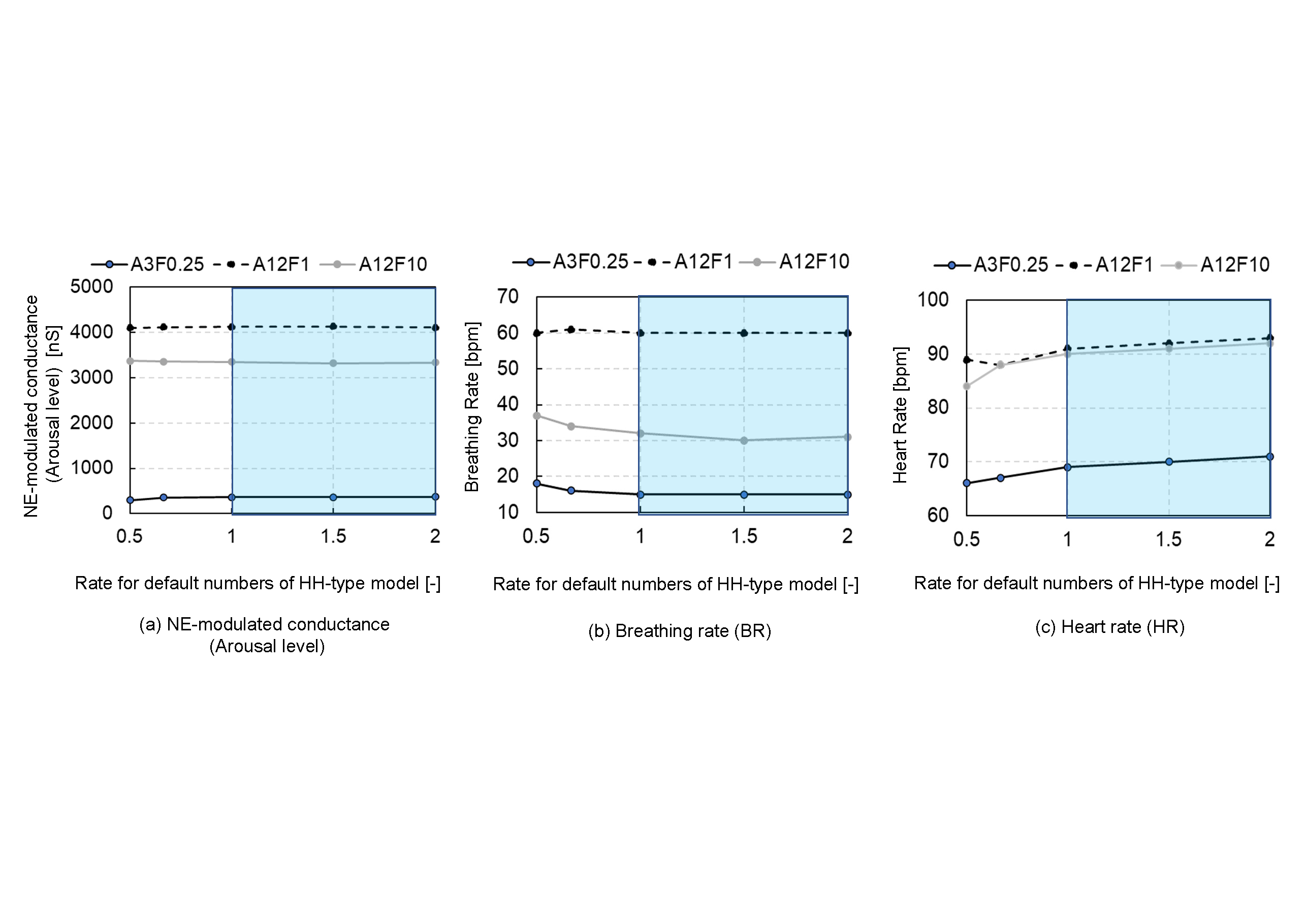

Supplement: Supplementary file 4 — Supplementary Figure S4. [file 41598_2023_32995_MOESM4_ESM.tif]
